# Development and iterative optimization of an independently usable assistance system to assess, maintain and improve the nutritional and mobility status of older adults: an iterative usability study

**DOI:** 10.1186/s12877-025-06950-1

**Published:** 2026-01-06

**Authors:** Mareike Förster, Lisa Happe, Vincent Quinten, Rebecca Diekmann

**Affiliations:** Department of Health Services Research, Junior Research Group ‘Nutrition and Physical Function in Older Adults’, Carl von Ossietzky Universität Oldenburg, Oldenburg, Germany

**Keywords:** Technical assistance system, Gerontechnology, Interactive health promotion, Technology optimization, Older adults, Independence, Nutrition, Mobility

## Abstract

**Background:**

Promoting a balanced diet and regular physical activity is crucial for maintaining independence in old age. Technical assistance systems can help identify nutritional and mobility deficits and initiate appropriate interventions. We are developing a technical assistance system consisting of a measurement and training station and a tablet-based app (AS-Tra). AS-Tra is specifically designed for independent use by participants aged ≥ 70 years to assess and improve their nutritional and mobility status.

**Objective:**

This study aimed to identify optimization potentials of the AS-Tra system together with members of the target group through iterative test cycles until good usability is achieved.

**Methods:**

The system is developed as a complex intervention in accordance with the Medical Research Council (MRC) framework and assessed in three iterative cycles for its usability. In each cycle, participants carried out structured predefined tasks. To capture their cognitive process, they were asked to ‘think aloud’. These thoughts were recorded with the necessary support for completing each task. Usability was measured using the System Usability Scale (SUS). Improvements based on the results were made. In the third cycle, independent use was simulated by leaving the participants alone in the lab, receiving tasks via audio instructions and observing them via webcams and eye-tracking glasses.

**Results:**

A total of 34 participants (78.60 ± 5.59 years, 70.93% female) were included in the three iterative cycles (C1–C3). The SUS score improved from 70.63 to 84.55 between C1 and C2 and decreased slightly to 78.18 at C3. Overall 63.11% of the tasks (*n* = 385) were completed without support. In 14.92% (*n* = 91) of all tasks, the task could be completed by repeating or rephrasing instructions, 20.33% (*n* = 124) with direct reference to the solution, and 1.15% (*n* = 7) of all tasks could not be completed independently.

**Conclusions:**

The results of the cycles made it possible to develop an independently usable system for assessing the nutritional and mobility situations of older people with good usability. Additionally, the relevance of support functions and the importance of research under real conditions became clear.

**Trial registration number:**

German Clinical Trials Register (DRKS), DRKS00031719, registered May 2023, https://drks.de/search/de/trial/DRKS00031719.

**Supplementary Information:**

The online version contains supplementary material available at 10.1186/s12877-025-06950-1.

## Background

Owing to ongoing demographic changes and the associated increase in life expectancy, the number of older people is growing significantly compared with that of the rest of the population [1].

Ageing is accompanied by age-related barriers, for example in the areas of cognition, physical activity and perception [2]. Muscle strength also decreases, which is why nutritional status plays an important role for older adults [3]. Maintaining and / or improving physical activity and a balanced diet are key factors in healthy aging and are essential for maintaining independence and preventing the need for long-term care [3, 4]. Healthy ageing can fundamentally be understood as a dynamic process that involves developing and maintaining the functional abilities that enable well-being in old age. At the heart of this process is the concept of intrinsic capacity, which was introduced by the World Health Organization. It refers to the totality of an individual’s physical and mental abilities. Recent studies on lifestyle interventions suggest that regular physical activity and a balanced diet can significantly support or even improve intrinsic capacity, thereby reducing the risk of needing care [5]. Regular monitoring of the two components mobility and nutrition of older adults often play a minimal role in care because of a lack of knowledge or too much time pressure [6]. Moreover, it is very unlikely that there will be enough nursing staff for the steadily growing proportion of the elderly population [7]. If physiotherapy or other therapies are initiated after an acute illness or hospital stay, it is quickly forgotten after returning home. The most common reasons for this are the engagement in exercise, low self-efficacy, medical problems such as pain with exercise, or other social and emotional factors that older adults face in their own homes. Lasting changes in behavior are rarely observed [8].

Regular monitoring of nutritional and mobility status can significantly contribute to the early detection of health risks and minimize negative health consequences. Such close monitoring is particularly important for older people living at home, as healthcare resources can be limited [9]. Regular monitoring can provide data on physical status, which, when combined with individual biological data, can provide clues to potential risks such as malnutrition [10]. For example, weight loss over time would be an indicator of malnutrition, and physical inactivity (determined by activity monitoring) would be a marker of functional decline and frailty [10, 11].

The use of assistive technology can enable independent measurements and exercise without medical staff. These complex interventions can be an approach to automatically detect health status and provide information to close knowledge gaps in nutrition and mobility management.

In general, technical assistance systems use newer technologies to support the target group in maintaining their autonomy to improve their quality of life. As technical assistance systems in the area of health care are often used in the home environment, they should also support the medical care process, e.g. by measuring the pulse or reminding people to exercise. A primary goal of these systems is early detection and thus preventive behavioral analysis to record indicators of changes in health [12]. The vast majority of technologies developed for older adults are mobile applications for smartphones or tablets that support for example, the regular intake of medication [13, 14] or the self-monitoring of nutrition [15, 16]. There are other health apps for tracking physical activity through the use of fitness trackers [17–19]. Complex interventions that take a multidisciplinary approach and cover several areas, such as nutrition and physical activity with the help of technological assistance, are not yet much to be found. There are apps such as NuMobe or KOKU-Nut [20, 21] which address both areas (nutrition and mobility), but lack the help of a technical system such as a measurement and training station. Furthermore, there are a few studies in which both exercise and nutrition are integral components in the development of a technical assistance system for older adults [22–26]. Independent use, especially in the home environment, is also the focus of [22–24]. A significant difference between the complex intervention described in this paper and the other studies is that, in addition to the app, a complete measurement and training station was developed, which provides geriatric assessments and additional training equipment for stability, reaction, and balance training. Furthermore, nutritional status is usually recorded indirectly [25, 26] and rarely through direct self-entry into a food diary or by completing integrated questionnaires. However, these systems could offer the opportunity to create an overall view of health status and thus contribute more comprehensively to quality ageing [27].

As it is often difficult for older people to access newer technologies, especially when they are alone, e.g., at self-scan checkouts in supermarkets and of course when using apps on touchscreens or tablets, their specific needs must be taken into account when developing such a system [28]. In addition, the effort and intensity of using technologies or systems to maintain the health of older people should be considered [29].

This paper describes the development and especially the iterative test cycles of the technical assistance system AS-Tra (Assistance System for sustainable improvement of nutritional and mobility status of older people (≥ 70 years) under consideration of the Transtheoretical model of health behavior change). The MRC framework was used to develop a complex intervention system. The first step focuses on the development of the system. The second step covers the feasibility after the development of the prototype. Under this point, the system presented here was first evaluated in iterative test cycles with the target group with regard to usability. The goal is a user-centered design focused on the specific needs of older adults, with the aim of supporting regular self-monitoring of mobility and nutritional status in order to maintain independence and promote healthy aging. Subsequently, a pilot study is planned in which the system is to be used by the target group over a longer period of time.

## Objectives

The aim of this study is, within the framework of a user-centered design approach, to precisely describe the iterative optimization steps of the so-called AS-Tra system with the measuring and training station and its app as a digital assistance system for seniors aged 70 years and over and thus to evaluate whether independent use can be reached by the target group.

## Methods

### Technical assistance system

The AS-Tra system was developed based on the phases of the MRC framework [30]. First, focus group discussions were conducted to determine which health data the target group was interested in and which technical devices they could imagine using. Potential barriers were also identified, for example, by asking the target group about their preferred time spent in the measurement and training station [31]. After that, the system was developed on the basis of the results and iteratively tested to assess its usability. In general, good usability can be defined as the extent to which users can achieve specific goals with effectiveness, efficiency and satisfaction [32]. The usability can be evaluated using the System Usability Scale (SUS) developed by Brooke et al. [33].

The technical assistance system consists of two main components: a tablet-based application and a measurement and training station. The station with its assessment and training devices measures physical function objectively. The app assesses nutritional status, provides information on nutrition and mobility, gives feedback to the user through a nutrition and physical activity diary, and displays the results achieved. The conceptual idea is that the app is used independently in everyday life and that the measurement and training station can be visited in a central place to perform measurements and training independently. To promote sustainable improvements in nutrition or mobility, the phases of the transtheoretical model for behavior change (TTM) [34, 35] are considered and used to individualize the contents of the app. This concept for describing, explaining and influencing behavioral changes comprises 6 stages, from the precontemplation phase (no intention to change behavior), through preparation (planning the behavior change) to maintenance or relapse [35]. This means for the app that when higher stages in the TTM are reached, new content is unlocked in the app that users were previously unable to see. This is intended to motivate users to continuously work on their nutrition and exercise status and prevents them from being overwhelmed.

#### Tablet-based app

The app was further developed based on the ‘NuMob-e’ app, previously developed as an age-adapted health electronic coaching system for older adults with deficits in nutritional status and physical activity, involving geriatric rehabilitation patients [20, 36, 37]. During the development of that ‘NuMob-e’ electronic coaching system the needs of older adults to improve their nutritional behavior and mobility status were identified through the focus group discussions mentioned above. As a result, the electronic coaching system considers different points of behavior change, offers obvious benefits, promotes motivation and enables adaptation to the individual patient [20, 36, 37].

For the development of the AS-Tra app, the functionality of the ‘NuMob-e’ app was ported from Android to Microsoft.Net MAUI Framework to enable it to run natively on both Windows and Android systems. In addition, integration with the measurement and training station and connections to the various assessments were implemented.

**Fig. 1:**
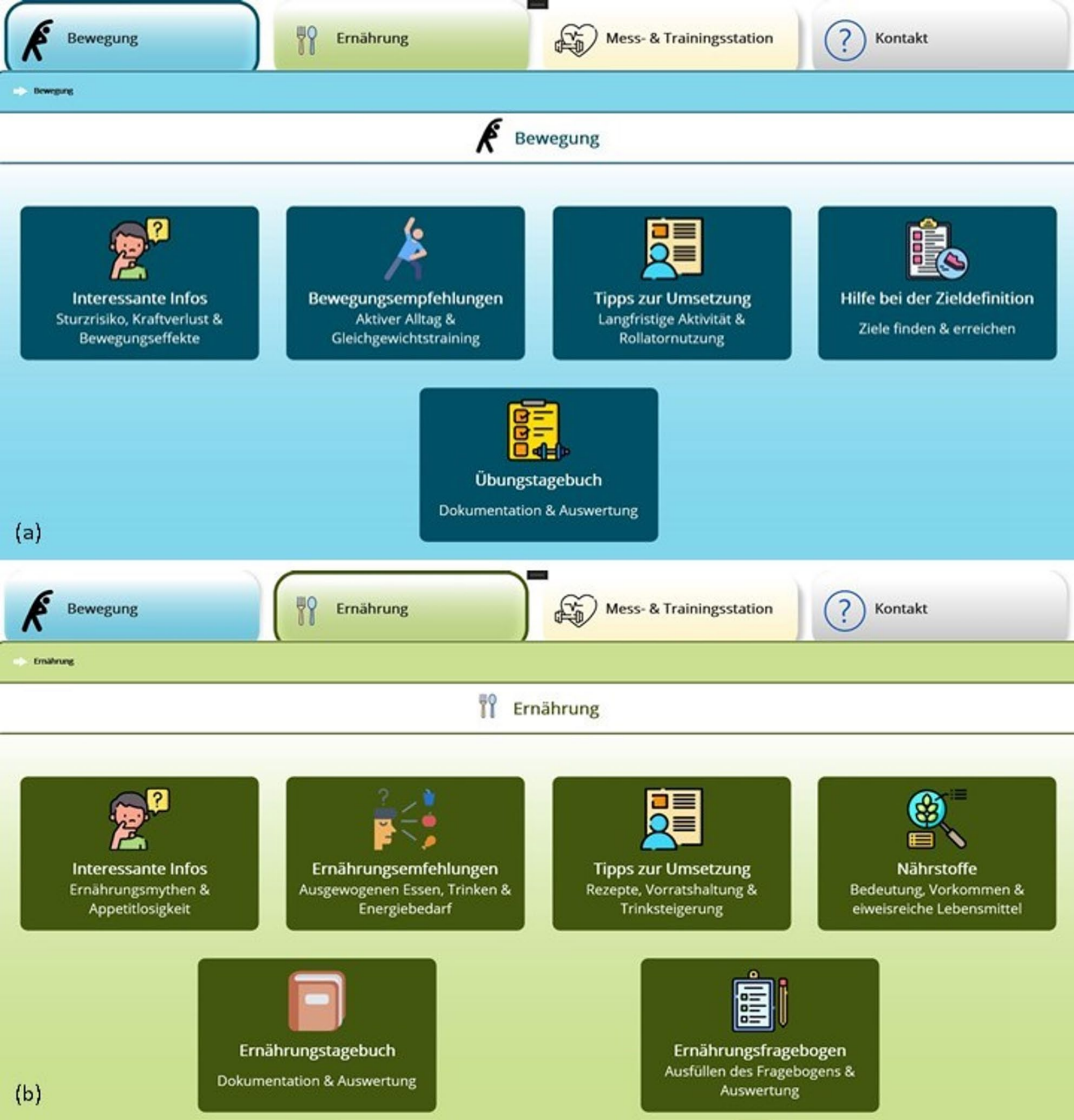
**a** Is a screenshot of the app interface on the topic of mobility with corresponding subtopics interesting info, exercise recommendations, tips for implementation, help with goal definition and exercise diary (from upper left to lower right). **b** Is a screenshot of the app interface on the topic of nutrition with corresponding subtopics interesting info, nutrition recommendations, tips for implementation, nutrients, food diary and nutrition questionnaire (from upper left to lower right)

Compared to NuMob-e’, the app-interface provides four instead of two different areas: (1) mobility, (2) nutrition, (3) measurement and training station, and (4) contact. In Fig. 1a, the mobility interface is shown in blue, and in Fig. 1b, the nutrition interface is shown in green, with their subtopics presented in colored highlighted tiles with corresponding icons. These tiles can be clicked to navigate to the subtopics. In the areas of mobility and nutrition, educational elements are built-in, such as texts, videos and quizzes, for example, physical activity recommendations, certain nutrients or nutritional myths [20, 36, 37]. Interactive elements include exercise and food diary, which enable older adults to record exercise and nutritional consumption. Feedback is provided, for example on consumed protein, drinking quantity or the amount and intensity of activity during the day. The MNA-SF [38] is also integrated to screen the risk of malnutrition and can be completed independently in the app.

#### Measurement and training station

**Fig. 2:**
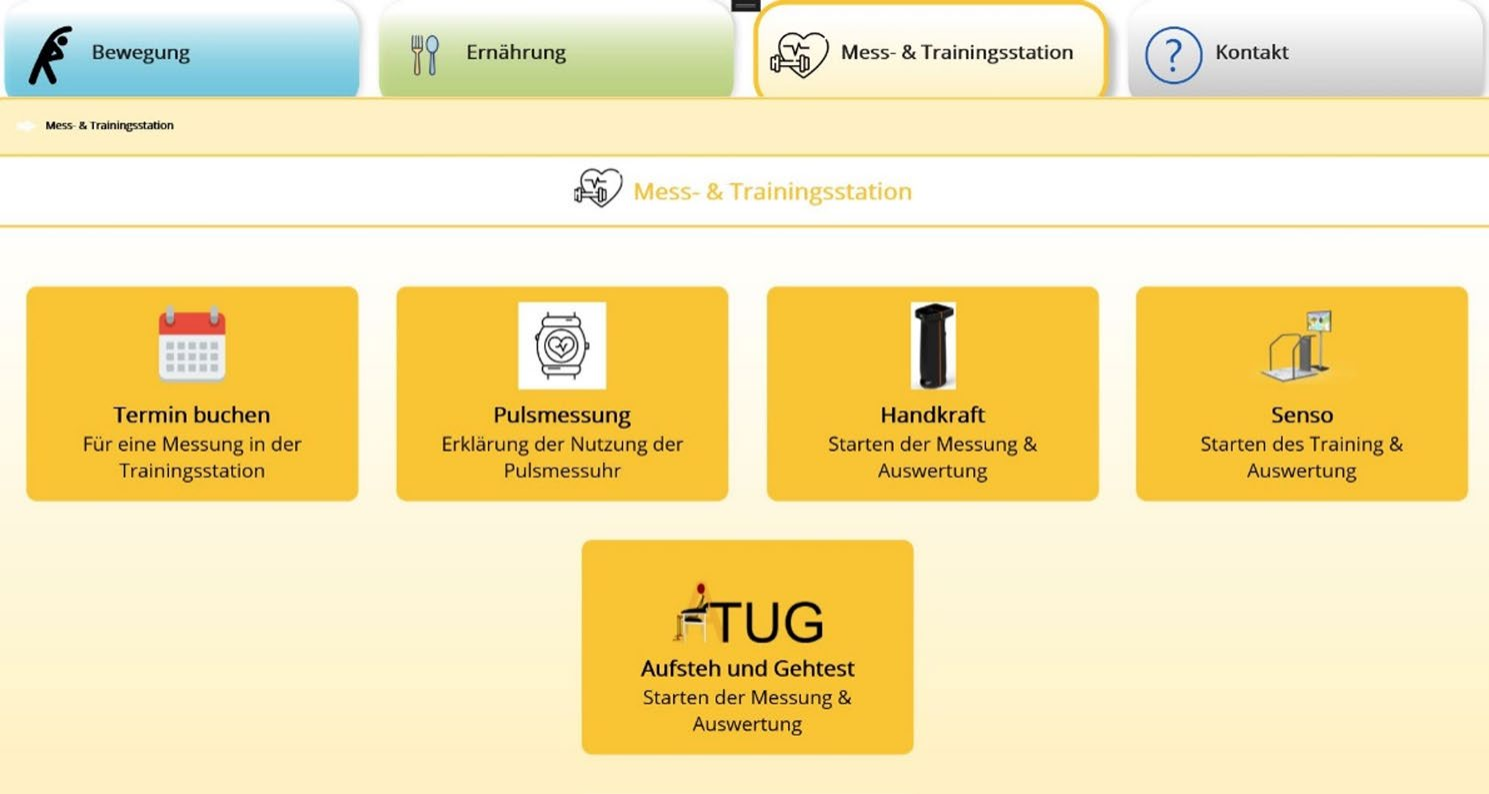
Screenshot of the app interface on the topic of measurement and training station with corresponding subtopics book appointment, pulse measurement, hand grip strength measurement, Senso, Timed ‘Up and Go’ test (from upper left to lower right)

The measurement and training station section of the app (Fig. 2) integrates different assessment and training devices used at the station. Figure 2 shows the measurement and training station interface in yellow with its subtopics (measurement of pulse, hand grip strength, exercise on Senso, measurement of Timed ‘Up and Go’).

**Fig. 3:**
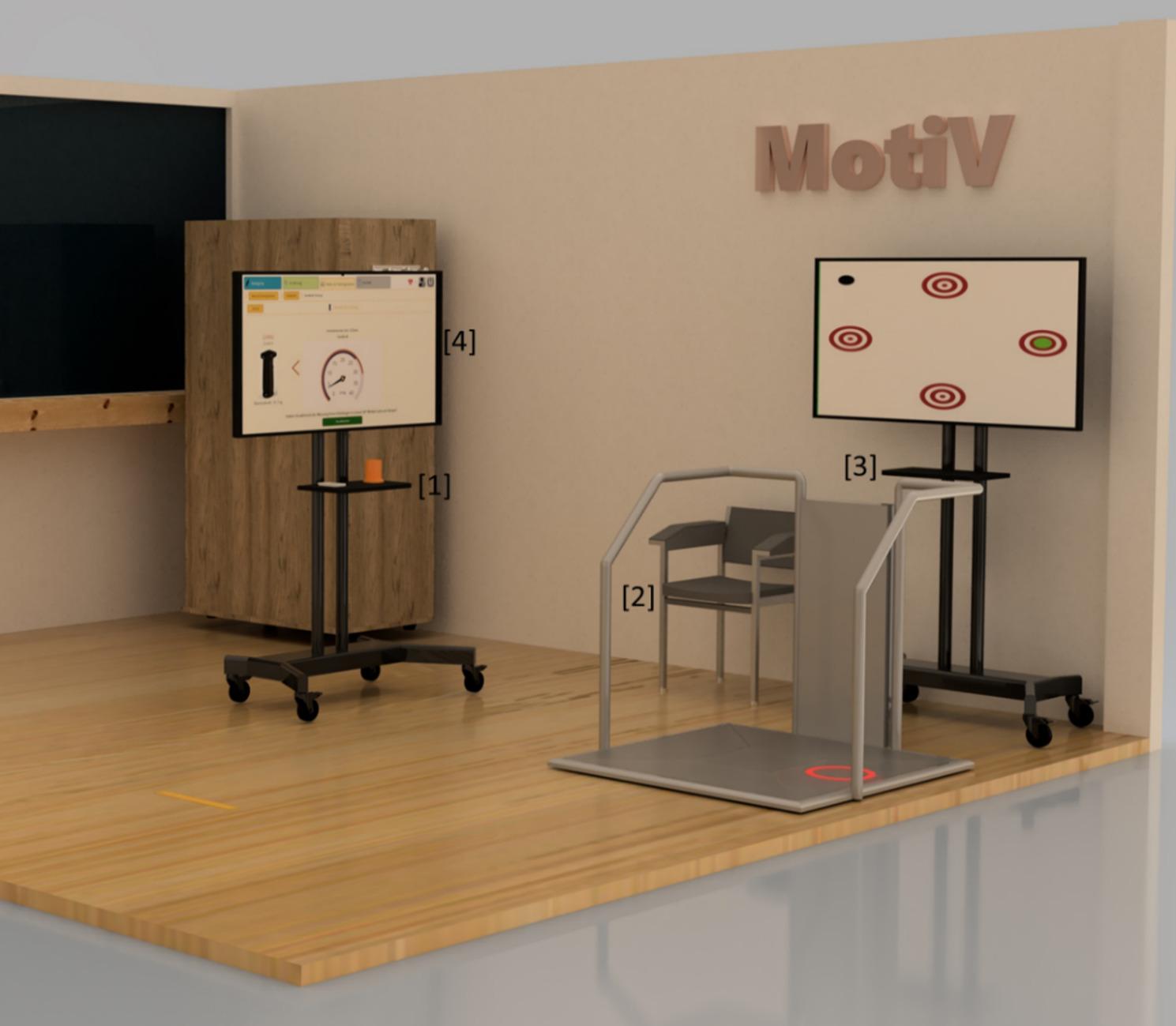
Schematic representation of the study room with [1] a table on which the Withings Scan Watch pulse measurement and the K-Force Grip Kinvent hand grip measurement devices are placed [2], the aTUG chair [3], the THERA-Trainer Senso training device and [4] the 55-inch touchscreen

The app runs on a Windows desktop computer, to which all devices are connected. It is located on site in the lab with a measuring and training station and uses a 55-inch touchscreen display (Fig. 3 [4]). By using the buttons in the app for the corresponding assessments or training, the user is guided through the whole process via textual explanations and explanatory videos. At the end of each assessment / training, the results are presented to the user.

**Fig. 4:**
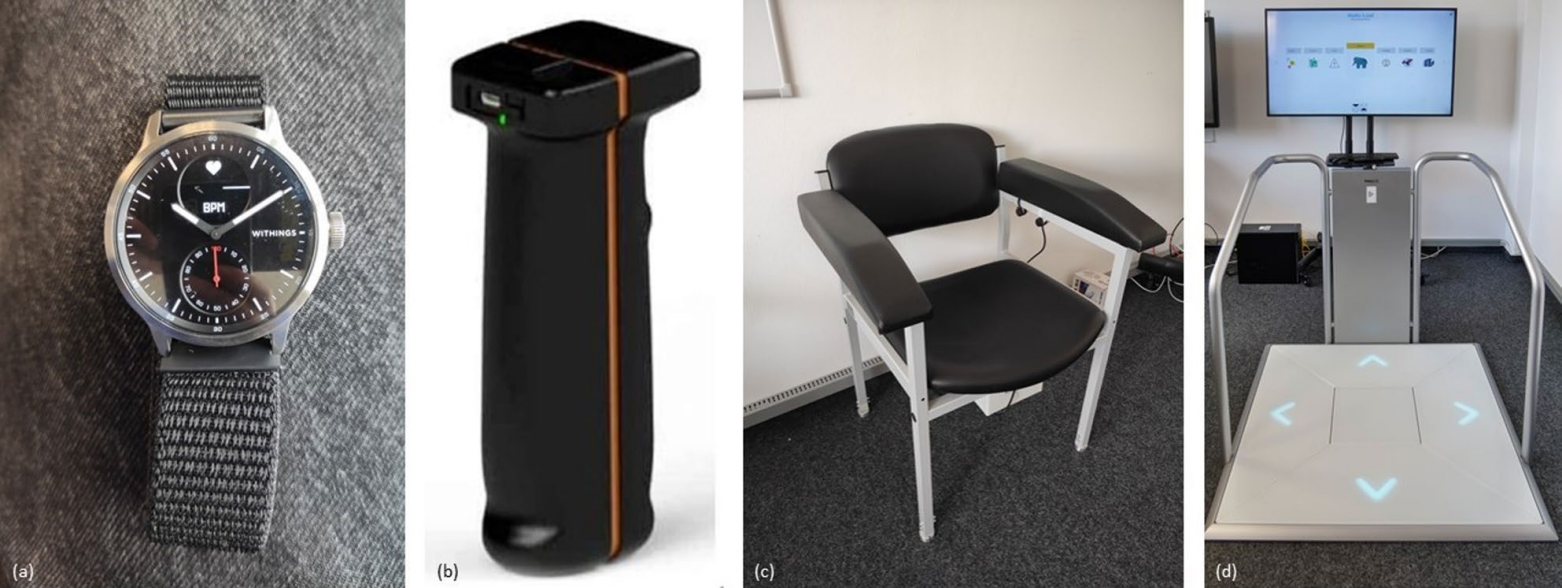
**a** shows the Withings Scan Watch for pulse measurement. **b** shows the K-Force Grip Kinvent for hand grip strength measurement. **c** shows the aTUG chair for the timed ‘Up & Go’ test and **d** shows the THERA-Trainer Senso for training at the measurement and training station

To monitor the pulse of the older adult during measurement and training, the Withings Scan Watch (Withings, Model HWA09) (Fig. 4a) is used. Hand grip strength is measured as predictor of functional deterioration [39] using the K-Force Grip (SAS Kinvent Biomechanique, K-Force Grip) (Fig. 4b). This device connects to the computer via Bluetooth and measures isometric hand grip strength in kilograms. The third assessment is the Timed ‘Up and Go’ test, which is associated with general mobility and the risk of falling [40, 41]. Therefore, the aTUG-Chair is used (Fig. 4c). This chair is fitted with infrared light barriers that automatically detect whether the participant is sitting on the chair and record the time needed for this test [42, 43].

The Senso (THERA-Trainer, model Senso 642) is used as training device (Fig. 4d). It has five force plates, one each at the center, top, bottom, right and left, to record the movement of the person on the device. Different cognitive and physical training programs can be selected using the accompanying ‘Dividat Play’ software. The training programs playfully train different areas such as coordination, balance and strength endurance.

The measurement and training results are displayed graphically and textually in the app. Therefore, each graph shows the results of the first measurement and the last three visits of the measurement and training station. This result display is integrated for all measurements (hand grip strength and aTUG) as well as for the training results on the THERA-Trainer Senso. This gives the participants an overview of whether they improved, remained stable or deteriorated compared to the previous and the first measurement.

### Participants

Participants were recruited via flyers and during specifically organized information events in community-based organizations, e.g. assisted living homes or public meeting places for older adults. In addition, potential participants (over 70 years old) were contacted via the AS-Tra project’s register of people interested in the project, informed about the study and invited to participate. Recruitment took two to five weeks per cycle, and the required number of participants was recruited within a total of ten weeks, with breaks between individual cycles. Recruitment took place continuously during the iterative cycles so that participants could be invited on an ongoing basis.

The age group of people over 70 was selected because it differs significantly from the group of people over 65, who are often still working [44]. The likelihood of health problems increases again with age [45] and this age group also lacks the digital support that is particularly important in terms of prevention [46, 47] In addition, some of the elements in preliminary projects were already developed specifically for geriatric patients (e.g., the app) who already had mobility deficits or deficits in dealing with technical elements, and for fit older adults over the age of 70 [20, 36, 37, 42, 43].

In sum, 65 participants were gradually recruited of which 16 had to be excluded directly due to lack of interest in the study, health problems or time constraints. The rest were invited and then screened against the inclusion and exclusion criteria for the study.

Older adults with at least one of the following inclusion criteria were eligible for inclusion in the study: (1) malnutrition according to the Mini Nutritional Assessment Short Form (MNA-SF) [38]; (2) presence of one or more of the following risk factors for malnutrition: weight loss within the last three months, decreased food intake in the last three months or a low Body Mass Index (BMI < 22 kg/$\:{\mathrm{m}}^{2}$); (3) the presence of signs of reduced mobility identified by a walking speed of < 0.8 m/s; and (4) Short Physical Performance Battery (SPPB) of < 8 points [48]. In addition, the participants had to be able to visit the study center.

The exclusion criteria were as follows: (1) the inability to consent; (2) insufficient ability to understand the study content, its process or the German language; (3) severe mobility limitations (unable to walk); and (4) severe visual limitations that prevented independent operation of the system (unable to see text and videos on the screen). The participants were not allowed to participate in more than one test cycle.

### Study design and procedure

The AS-Tra system described above was evaluated with older adults in three iterative test cycles (C1–C3), whose study design and procedure are explained in more detail below. In general, the method applied here is based on a cyclical usability optimization process. In three cycles, the system was iteratively adapted to the needs of the target group. This is based on the structured analysis of task solutions through defined usability tasks and the assessment of the necessary level of support (e.g., repetition of the task, reformulation, demonstration). Furthermore, the “thinking aloud” method was used throughout, allowing users to provide real-time feedback on comprehension difficulties, implementation issues, and suggestions for improvement. This feedback flows directly into the next version of the system, allowing for continuous adaptation and further development to improve usability. The individual usability tasks and the setup for each iterative cycle are described in detail below.

Each cycle included at least ten participants in accordance with the recommendations for usability tests of Faulkner [49] who were recruited anew in each cycle and each participant was only allowed to take part in one cycle. After informed consent was obtained, the inclusion and exclusion criteria were checked. This was followed by the collection of sociodemographic data and technology commitment. Neyer and Felber’s scale was used to measure technology involvement. This questionnaire uses a 5-point Likert scale with a total of 12 items covering statements on interest, use and attitudes toward technology [50]. It applies to all everyday technologies (e.g., microwave, television, cell phone, etc.) and is completed by participants on-site. Afterwards, the usability test described below was then carried out. When a participant had finished the usability tests, the usability was evaluated last using the System Usability Scale [33]. For the classification of the SUS score, the adjective rating scale of [51] was used. A SUS score of 25.10–51.60 describes a tested system as ‘poor’ usability, from 51.60 as ‘ok’, from 71.10 the usability is classified as ‘good’, from 80.80 to 84.00 as ‘excellent’ and from 84.00 as ‘best imaginable’. All data collection was carried out in-person and on-site testing. Each of the participants went through the process alone. there was never more than one participant and the study staff in the measuring and training station.

#### Usability tasks

Before the usability tasks were started, the test procedure was explained and the participants were briefly introduced to the system, including its contents and navigation options. During this introduction, it was emphasized that the aim of the study was not to test their abilities but to test the usability of the system.

For the iterative test cycles, usability tasks were defined and carried out in the areas of navigation and comprehension. The navigation tasks were used to check whether the older adults could find their way through the app to the predefined topics, whether they could use the buttons correctly and whether they could start and carry out the various measurements independently at the station. The comprehension tasks were intended to check whether the content was interpreted correctly or whether the information was understood correctly.

The entire test was divided into several larger sections and each individual section is color-coded, which corresponds to the more detailed test protocol in the appendix and is intended to make it easier to assign the tasks to the individual sections when comparing these two tables. These sections were defined based on the respective measurements and training sessions, for example, there was one section for grip strength measurement or one for training on the THERA-Trainer Senso. In each of those sections, there were several tasks, mostly from both areas (navigation and comprehension). These individual tasks can be assigned to different categories. In the navigation section there were 3 tasks that belong to simple navigation, e.g., you had to navigate in depth a maximum of twice. 3–4 tasks were assigned to complex navigation, where you had to navigate in depth more than twice. In the comprehension area 5 tasks were assigned to the category use of elements, 4–5 tasks belonged to the category interpretation of test/training results and 1–2 tasks were assigned to the interpretation of the subject areas in the app. Owing to the slightly different test logs of the iterative cycles, there were different numbers of usability tasks in some categories.

The elements that were examined as part of the iterative testing are listed in Table 1. For more details of the individual color-coded section, the whole test log can be read (see Appendix).

**Table 1:**
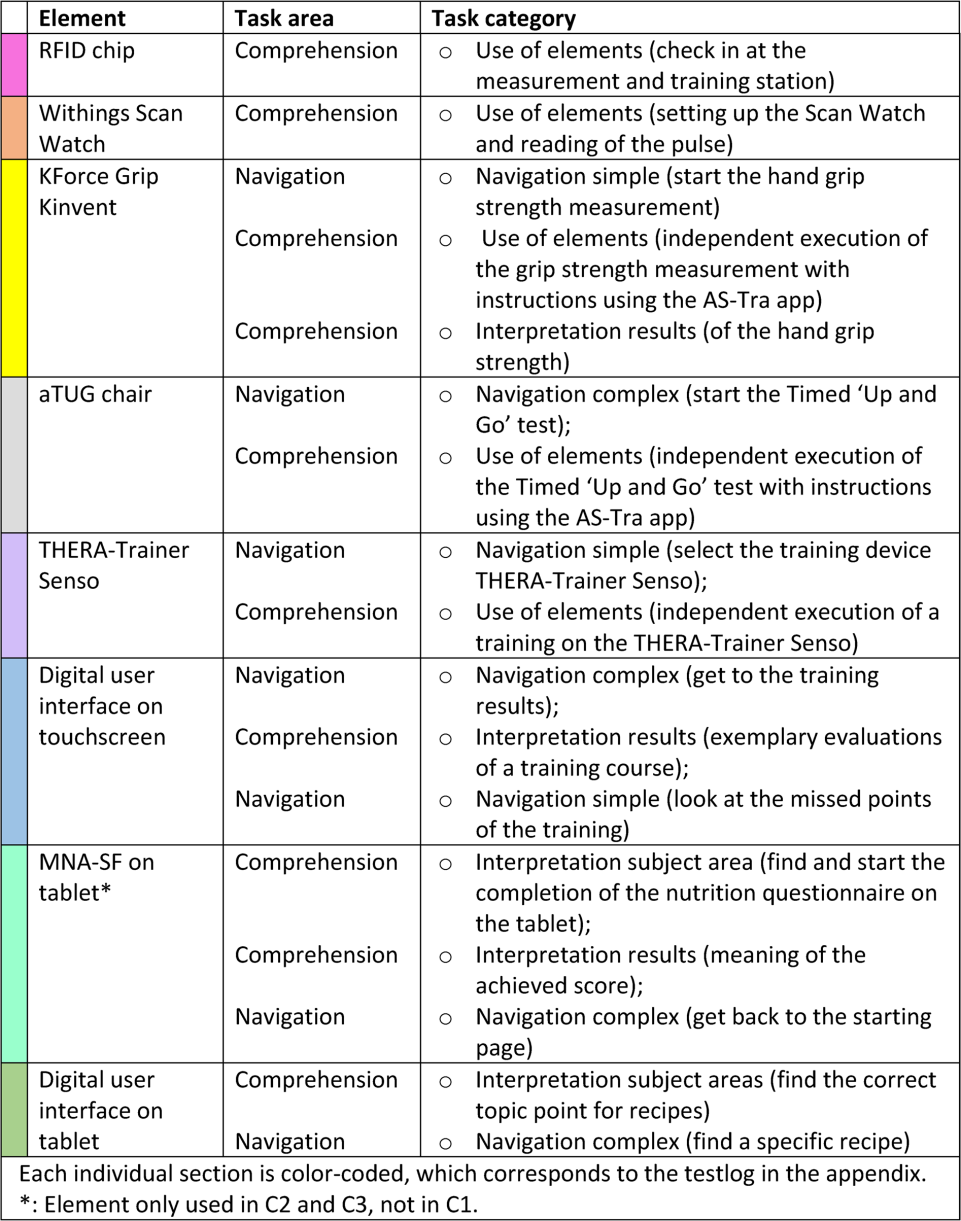
Elements used and corresponding tasks are categorized according their area and then more precisely according to the respective category in this area. The order of the elements used corresponds to the order in the iterative tests

During the test, all tasks were observed by two members of the study staff. One person conducted the test and provided the tasks and help (if necessary), and the other person timed the test and took notes. The study staff did not interrupt the participants during the testing, unless the participant asked directly for help or was obviously experiencing difficulties. The help provided by the study staff was recoded into three different levels: Help (1) repetition or rephrasing of the task at hand; Help (2) providing direct help to the solution; Help (3) the participant being unable to complete the task at hand and the study staff having to intervene.

The participants were instructed to apply the ‘Thinking Aloud’- method during the test. These verbal comments and required assistance were documented on paper together with the time needed to complete each task.

#### Iterative process

A total of three iterative test cycles were performed. The iterative optimization process requires precise documentation using the think-aloud method. All problems that arise (for example, when using the assessment devices or completing usability tasks) are documented in detail in writing. Furthermore, everything the test subjects say during testing is recorded. In combination with the documentation of the assistance required, it is possible to determine which tasks presented the most difficulties, and the verbal comments often provide insights into the cause or even possible improvements. The iterative approach has the advantage that these improvements are integrated into the system after each cycle. This way, in the following cycle, based on the number of difficulties encountered and the test subjects’ statements, a direct comparison can be made as to whether the adjustments were helpful and whether any new problems have arisen, or whether the same difficulties still exist.

Regarding the setup, the first two cycles were identical. The app and the integration of the station were optimized through iterative testing. In the second cycle a nutrition questionnaire was added as a task (see Table 1 ‘MNA-SF on tablet’), which was completed independently on the tablet. Otherwise, the process and the tasks remained the same as those in the first cycle.

However, in the third cycle the setup was changed to investigate whether the participants could perform the tasks without the physical presence of study staff. For this purpose, the participants wore an eye-tracking camera (Tobii Pro Glasses 2, Tobii AB) and were observed by three webcams (1x RAPOO, XW180 Full-HD Webcam und 2x LOGITECH C922 Pro Full-HD Webcam) during the test. The eye-tracking camera was used to examine what content the participants were looking at and the webcams helped to observe the position of the participants in the room and how they performed the tasks (e.g. the Timed ‘Up and Go’ test). Instructions were given via audio and the participants were able to talk to the study staff through microphones.

### Data analysis

The software SPSS (version 29.0.0) was used for the statistical analyses.

Participants’ characteristics, the data of the MNA-SF, the SPPB, the technology commitment and the SUS were analyzed descriptively. The data are presented as total numbers, percentages and means with standard deviations. The usability tasks were also analyzed descriptively according to solved and unsolved tasks as total numbers and percentages for all older adults performing the task. In addition, the total number of hints required and the type of hint (Help 1, Help 2 or Help 3) were recorded.

Qualitative data were documented on paper using the ‘Thinking Aloud’- method to understand the cognitive process of the participant during the execution of the tasks. These notes were discussed by the study staff to identify ambiguities or optimization potential.

## Results

### Characteristics of the included participants

A total of 45 older people agreed to participatein the study. After screening for inclusion and exclusion criteria, *n* = 11 had to be excluded (*n* = 4 in C1, *n* = 2 in C2 and *n* = 5 in C3). There were 34 older adults (24 females, 10 males) with an average age of 79 years who participated in the iterative testing of our system. The first iterative cycle was carried out in June and July 2023, the second in September and October 2023 and the third cycle took place in February 2024. Table 2 lists the results of the MNA-SF and SPPB as well as the mean age, use of walking aids (e.g., cane or walker) and the mean technology commitment score of all three iterative cycles (C1-C3). The technology commitment in C2 was somewhat lower than that in C1 and C3. The mean value across all three iterative cycles is 41,10 ± 8,58.

**Table 2:** Characteristics of the included participants and the results of the MNA-SF, SPPB and technology commitment questionnaires are given in total numbers and percentages

		C1	C2	C3
Participants, total (*n*)		12 (7 female, 5 male)	11 (7 female, 4 male)	11 (10 female, 1 male)
Age (years), mean (SD)		77 (3.94)	80 (6.47)	79 (6.36)
Walking aids, *n* (%)		1 (8.33)	4 (36.36)	5 (45.45)
MNA-SF^a^, *n* (%)	Normal	3 (25.00)	6 (54.54)	4 (36.36)
	Risk	9 (75.00)	5 (45.45)	6 (54.54)
	Malnutrition	0 (0.00)	0 (0.00)	1 (9.09)
SPPB^b^, points (%)	0–4	0 (0.00)	0 (0.00)	0 (0.00)
	5–8	4 (33.33)	5 (45.45)	8 (72.72)
	9–12	8 (66.66)	6 (54.54)	3 (27.27)
TC^c^, mean score (SD)		42.75 (7.83)	37.27 (10.49)	43.27 (7.43)

^a^MNA-SF: Mini Nutritional Assessment Short Form

^b^SPPB: Short Physical Performance Battery

^c^TC: Technology commitment

### Usability tasks and SUS

The tasks in the attached test protocol were divided into individual colored sections as shown in Table 1, for which the times were measured as to how long it took to complete the sections with the corresponding tasks.

The total mean time needed for a whole test in C1 was 23.08 min, with a standard deviation of 2.35 min. In C2 the time was 25.17 ± 08.10 min and in C3, an average of 21.34 ± 04.53 min was needed. The time required for pulse measurement using the Withings Scan Watch in C3 was, on average, approximately twice as long as in the first two iterations. On the other hand, in C1, approximately twice as much time as in C2 and C3 was needed for the use of the digital interface on the tablet. The other times of the individual sections did not differ greatly from each other. There was no time for the MNA-SF on the tablet of C1 because the MNA-SF was added in the second cycle.

**Fig. 5:**
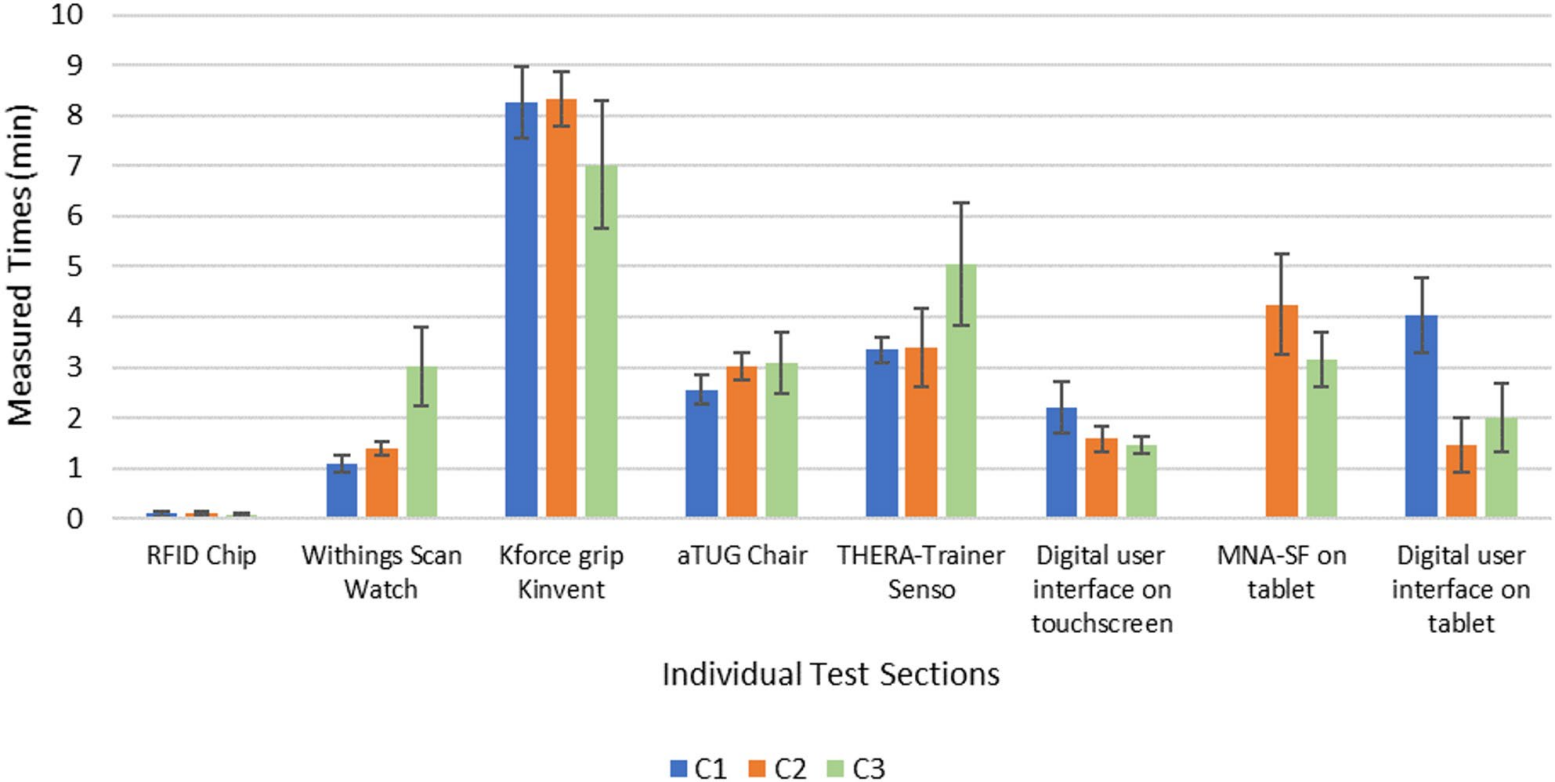
Times needed for the individual sections of the tasks in minutes. The results of C1 are shown in blue, those of C2 are shown in orange and those of C3 are shown in green. Standard deviations are also marked

These mean times are shown with their standard deviation in Fig. 5.

The SUS score, as well as the number of tasks completed, including the support needed, divided into navigation and comprehension tasks, are summarized in Table 3. The SUS score increased from 70.63 in C1 to 84.55 in C2. The value in C3 then decreased slightly to 78.18.

**Table 3:** SUS score, total number overall, number of navigation and comprehension tasks and a breakdown of how many of the tasks needed, which help in each case for all three iterative test cycles C1 to C3 are summarized. Missing information is also noted

	C1	C2	C3
Navigation Tasks, *n* (%)	72 (37.50)	77 (36.84)	77 (36.84)
Total number of tasks, *n* (%)	192 (100.00)	209 (100.00)	208 (100.00)
No Help, *n* (%)	42 (21.88)	59 (28.23)	49 (23.44)
Help 1^a^, *n* (%)	12 (6.25)	10 (4.78)	10 (4.79)
Help 2^b^, *n* (%)	15 (7.81)	8 (3.83)	18 (8.61)
Help 3^c^, *n* (%)	2 (1.04)	0 (0.00)	0 (0.00)
Missings, *n* (%)	1 (0.52)	0 (0.00)	0 (0.00)
Comprehension Tasks, *n* (%)	**120 (62.50)**	**132 (63.16)**	**131 (63.16)**
No Help, *n* (%)	73 (38.02)	82 (39.23)	80 (38.28)
Help 1^a^, *n* (%)	10 (5.21)	28 (13.40)	21 (10.04)
Help 2^b^, *n* (%)	34 (17.71)	22 (10.53)	27 (12.92)
Help 3^c^, *n* (%)	2 (1.04)	0 (0.00)	3 (1.44)
Missings, *n* (%)	1 (0.52)	0 (0.00)	1 (0.48)
SUS score, mean (SD)	**70.63 (17.42)**	**84.55 (13.55)**	**78.18 (12.55)**

^a^Help 1: Repetition or rephrasing of the task at hand

^b^Help 2: Provide a direct help of the solution

^c^Help 3: The participant is unable to complete the task at hand and the study staff have to intervene

In C1, 192 tasks were performed in total (16 per participant), of which four tasks could not be completed independently by the participants. Two of them were navigation tasks and two were in the area of comprehension. The navigation tasks where the study staff had to intervene were, on the one hand, complex navigation to the result page of the training, on the other hand, simple navigation to another tab on the same page. One comprehension task that could not be solved independently was in the category interpretation of measurement/training results. The second was in the category of use of elements. The element that was difficult to use was the aTUG chair. With respect to the tasks where direct help to the solution was needed, it was most difficult to complete a complex navigation on the tablet.

During C2, a total of 209 tasks were performed, including the task of completing the MNA-SF on the tablet. All tasks were completed by the older adults, and less direct help was needed (8 instead of 15 in the area of navigation). Regarding the navigation tasks, it was again most difficult to complete simple navigation. Some participants also struggled with the complex navigation of the element hand grip strength measurement. In the area of comprehension, most of the assistance needed to use the element hand grip strength measurement. in addition to C1, complex navigation on the tablet was difficult and moreover, understanding the structure of the MNA-SF was also difficult.

In C3, a total of 208 tasks were performed (1 was missing), and three tasks could not be completed independently, all of which were in the area of comprehension. These tasks were as follows: twice, use the element for pulse measurement and once, use the element for hand grip strength measurement.

In C1, 59.90% of all tasks could be solved independently; in C2, 67.46%; and in C3, 61.72% of all tasks were solved without any help.

### Technical functioning

When dealing with the technology, two main errors occurred during the iterative cycles: First, the Withings Scan Watch was often difficult to use. This signifies that there were problems when manually switching on the Scan Watch and when starting the pulse measurement on the Scan Watch. Second, some participants had problems using the touchscreen at the station. In all three test cycles, a general observation was that the touchscreen did not respond well for some older adults. This error was dealt with in different ways. Some participants simply tried to tap more often using their fingers, whereas others used the touch pen directly, which was offered after C2. Therefore, an observation was, that even the touch pen did not necessarily improve the touch of the screen.

### Optimization of the app

With respect to the optimization of the measurement and training station with the AS-Tra app, the hardware was adapted (especially when changing the setup in C3) or extended (e.g., by the MNA-SF or the touch pen). With the help of the ‘Thinking Aloud’ comments, the navigation structures were also adapted and textual, auditory and visual elements were added for better comprehension. The adaptations from C1 to C2 and from C2 to C3 are listed in Table 4.

**Table 4:** Optimizations of the app from C1 to C2 and from C2 to C3 according to the terms Hardware, navigation and comprehension are indicated here

	Adaptations from C1 to C2	Adaptations from C2 to C3
Hardware	o Replace wrist band of Scan watch with one with Velcro o Add nutrition questionnaire (MNA-SF) to fill out independently on the tablet	o Offer a touch pen o Create all tasks and instructions as audios o Installing cameras and microphones for the setup without physical presence of study staff in C3 o Use of an eye-tracking camera for the setup without physical presence of study staff in C3
Navigation	o Change of the starting pages, when opening the app o Add subtitles o Larger tiles with icons and larger font size	o Adaptation of the layout of the MNA-SF to make it easier to select possible answers and change answers from previous questions if necessary
Comprehension	o Add instruction video for the aTUG with detailed explanation of use o Add more instructional text during hand grip strength measurement to describe the use step by step o Adaptation of the graphical representation of the results to highlight the most recent measurement and add more detailed description of this graphic o Reference in text to missed points for training results of the THERA-Trainer Senso	o Add instruction video for pulse measurement with detailed explanation of use

## Discussion

The aim of this study is, within the framework of a user-centered design approach, to precisely describe the iterative optimization steps of the so-called AS-Tra system with the measuring and training station and its app as a digital assistance system for seniors aged 70 years and over and thus to evaluate whether independent use can be reached by the target group.

### Principal results

A total of three iterative cycles of testing were carried out. A comparison of the three cycles revealed that the usability of our assistance system with the AS-Tra app could be improved. The chosen methodology, consisting of a test protocol with clearly defined usability tasks in the area of ​​navigation and comprehension, the documentation of required assistance and the time required to complete the tasks as well as the use of the ‘Thinking-Aloud’ method, helped to develop an interactive technical assistance system. From C1 to C2, the usability score increased from 70.63 to 84.55, and all tasks in C2 could be completed independently by the older adults, which showed that less help from the study staff was needed to complete the tasks. One reason for this could be the adjustments made to the app after the first cycle. The optimization after each iteration by e.g. the addition of subtitles, instructional videos and texts, and adjustments to the graphical representations of the results (see Table 4) may have led to a situation where light assistance (Help1 or Help2) was sufficient, allowing participants to complete the tasks independently.

The SUS score from C1 indicated that the usability of the AS-Tra system was ‘ok’ in the first cycle. In C2 the usability was ‘best imaginable’ at 84.55 and from C2 to C3, the SUS score was 78.18, indicating that the system was considered ‘good’ [51]. A comparison of these results with those of C3 revealed that the SUS decreased slightly to 78.18 and that three tasks (1.44%) could not be completed despite help, whereas help was needed for comprehension tasks. This lower usability could probably be attributed to the different settings, as the participants were alone in the room without the physical presence of study staff during C3 [52]. described in their review the importance of support for older adults in web-based interventions to promote healthy lifestyles. In particular, human interaction has been emphasized. However, there is a wide range of what older adults want in terms of support, ranging from purely web-based to face-to-face counseling and training with a coach [52]. In an article that also used exergames to rehabilitate older adults, the importance of interpersonal interaction and human contact was emphasized [53]. This signifies that the setting in which older adults were left to their own during testing and training could have an impact on their well-being and could be a reason for slightly poorer usability. As an example, in the present study, the auditory indication of tasks in C3 could be a problem, as older adults are sometimes hard-of-hearing; particularly high or particularly low frequencies are then difficult to understand [54]. The fact that the audio was played through loudspeakers could lead to comprehension problems and thus also affect usability. This problem was counteracted by adding subtitles to the explanatory videos.

Nevertheless, the change in the SUS score between C2 and C3 underlines the relevance of investigations under real conditions and can also be interpreted as an indication that the current system design is not yet sufficiently adapted to the requirements for fully autonomous use by older adults.

Other previous works that used the System Usability Scale in the development of technical systems with older adults also achieved ‘good’ usability. An iterative study, a case risk mHealth app for older adults, achieved a score of 84 when used on a smartphone and a SUS of 80 when used on a tablet [55]. In another pilot study, a swallowing training app for older adults was tested in terms of usability and feasibility. The SUS score of this app can be classified as ‘ok’ at approximately 70 [56]. Another technical fall prevention system, which investigated the user experience and technical acceptance in a randomized clinical trial, also used the SUS and achieved a usability of 62 [57]. All of the previously mentioned studies focused on older adults aged 65 years and over, which means that the average age was somewhat younger than that in the present study. All studies focused on the testing and usability of technical systems (mostly apps) for this target group. The main difference between the study presented in this paper and previous works is that the AS-Tra system is multidisciplinary, covering multiple domains (mobility and nutrition), including the independent use of measurement and training devices, and does not consist of just one app, which is why it is a complex intervention. Most health apps and systems for older adults are related to one area of health, with mobile and web-based technologies being the most widely used apps [27]. Due to the variety of assessments, methods and different focuses used (e.g. managing daily activities, supporting healthy lifestyles, physical activity or nutrition), many perspectives must be considered, and many areas must be linked to develop complex, technical systems for older adults.

From the notes taken during the tests, it was striking that problems occurred during all three iterative cycles with the use of the element Withings Scan Watch to measure the pulse. These and other problems with integrated assessments often occurred because the instructional videos were not watched until the end or because the instructional texts were not read. The older adults usually tried to perform the assessments by themselves while the explanatory video was still running. They were then often not fast enough to perform the task in parallel with the video or were surprised that they had to do the same thing again after the video ended. In the study, an attempt was made to avoid this by inserting text above the videos indicating that the video should be watched to the end and that nothing should be done yet. Additionally, this message was integrated into the beginning of the video as audio and text and into the task itself. However, the subsequent testing was not affected, except that some participants were briefly confused about having to perform the test again.

To the best of the authors’ knowledge, there are no separate studies that address this problem of the inattention or impatience of older adults during self-assessments [58]. stated that in their study about the feasibility and acceptability of an interactive ICT platform on a tablet for managing patient-reported concerns for older adults at home, there were no problems submitting reports as instructed. However, only half of the participants read the self-care advice and the others forget that they have access to it. The reason for this was that the use of tablets was new, but how the problem could be solved has not been discussed [58]. A study by [59] on the implementation and validity of a cognitive assessment tool for older adults revealed that participants had no problems following the instructions and performing the tasks [59]. A pilot study on home-based self-delivered prehabilitation interventions to proactively reduce the fall risk of older adults by [60] also presented no results on impatience but address the visualization techniques used and their effectiveness [60]. Similarly, all the usability studies mentioned above do not refer to this specific problem. The only study where this behavior of not reading or watching the instructions to the end could also be observed is that of (51), during the ‘Self-TUG test’. The researchers tried to fix it with a pop-up warning message, but a bug prevented this from occurring in most of the tests. This means that they could not make a clear statement about the effectiveness of such warnings in the app. Therefore, it seems that there is no published solution to the problem yet.

During the tests, using ‘Thinking-Aloud’, the participants indicated that it would be helpful for them when additional information is provided on the buttons so that they know what to expect when clicking the button. Two examples for that are once the ‘Senso’ button in the measurement and training area (see also Fig. 1c). Here, ‘Senso’ is the title, and the added subtitles are ‘start training & evaluation’. In the nutrition section, the subtitle ‘recipes, stockpiling & increasing hydration’ has been added to the ‘Tips for implementation’ button (Fig. 1b) to clarify what is behind it. After adding these subtitles for C2 (Table 3), less direct help was needed in the area of navigation, from 15 (7.81%) tasks in C1 to only 8 (3.83%) tasks in C2 (Table 2). Furthermore, the time needed to perform a complex navigation on the tablet and search for a specific recipe was halved from C1 to C2 and C3, which may also be related to the subtitles. What is also striking is that the total time required for all usability tasks seems to be significantly higher in C2 than in C1. However, the times required for the individual sections did not differ significantly (see Fig. 5). The major difference is in the task of completing the nutrition questionnaire. Since this task was not yet available in C1, this time is added to the time required for each participant in C2, significantly extending the overall study time.

In addition, participants often stated that a motivating factor is very important to them, i.e., that they could also see whether they improved in the measurements and developed more grip strength, for example. This information is consistent with the literature. A recent review highlighted that the aspects of digital technologies for older adults that can promote their independence include self-monitoring, personal feedback and goal setting, personalized training and gamification [27, 61].

All of these minor adjustments in design or general input from older adults to improve the system for their personal needs play an important role in developing an assistance system for this particular target group. As [56] mentioned, identifying usability issues relevant to the daily lives of older adults would be helpful if the studies were also conducted with this target group. The review of [27] also revealed that early and close involvement of older adults in the development of assistance systems is important in order to counteract the lack of trust in technologies among older adults.

Another observation that influences usability is that the touchscreen does not respond well. The design of the buttons was identified as a possible reason for this. According to [62], older adults are more likely to press an icon than a button with text. In addition, the skin of older adults is often drier and more leathery, which can mean that the electrical charge does not change enough when touching a touchscreen [63, 64]. However, since the use of the touch pen did not necessarily lead to an improvement in this problem, it may also be due to the interpretation of the touch by the screen, as older adults tend to often press buttons for longer, the screen might not recognize it as a click event [65]. For further use, different settings of the touchscreen are tested to determine whether the screen reacts more sensitively with certain settings, and this also perceives touches with dry fingers, for example. In addition, a different package and design will be used for the buttons to ensure that the icons on the buttons have no influence on the touches.

### Limitations

Although the participating older adults had to have a deficit in nutrition and/or mobility to be included in the iterative study, it is likely that the participants who signed up already had a general interest in physical activity and nutrition or in technology. Therefore, a selection bias cannot be ruled out, even if at least the technology commitment also corresponds to the numbers from the study of [65] with older adults in Germany with varying degrees of experience in the use of technology [65]. Another limitation is that cognitive impairments, along with visual impairments and insufficient ability to understand the course content and the German language, were not included as a separate exclusion criterion. The MNA-SF includes a question regarding cognitive impairments, but it should be kept in mind that cognitive difficulties can generally have a strong impact on technology use. This means that whether participants are able to understand the study content is a subjective assessment by the study team. However, this assessment is supported by the objective assessment of health status using the questionnaires used (MNA-SF, SPPB).

Additionally, the changed setup in C3 may have biased the results because the participants could feel left alone and because there were no study staff members in the room, there was also no direct, obvious contact person to ask for help. Moreover, this study could only be used to make statements about usability for a single use. Nothing can be said about the change in usability over a longer period of use and the effectiveness of using the AS-Tra for nutrition or physical activity.

The data were collected by study team members who were themselves involved in the development of the AS-Tra system, which does not provide the same objectivity as if a noninvolved person had conducted the study.

## Conclusion

This iterative study involved older adults to optimize an independently used technical assistance system that consists of a measurement and training station and an app. It has been shown that this approach leads to good usability of the system for the target group. The involvement of older adults is very important for properly addressing their needs and developing an adapted system. In addition, the relevance of support functions became clear by testing the independent use of the measurement and training station with the AS-Tra app without a member of the study team in the room, which also shows how important it is to investigate under real conditions.

Overall, this iterative study identified the optimization potentials with the help of older adults and thus developed an independently usable system with good usability. According to the results of the SUS, most older adults stated that they could easily imagine using the system regularly. Finally, the AS-Tra system is one of the first complex interventions with a multidisciplinary approach which combines technical assistance in the form of a measuring and training station including automated geriatric assessments, a training device and an associated app and can therefore provide a more complex and comprehensive overview of health status.

The monitoring of the mobility and nutritional status of older adults via the AS-Tra system and whether its use can contribute to an improvement as well as the acceptance and experiences in the long-term use of the system must be tested in further studies.

## Electronic Supplementary Material

Additional file 1.

## Data Availability

The data generated during the current study are available from the corresponding author on reasonable request.

## Abbreviations

AS-Tra: Assistance System for sustainable improvement of nutritional and mobility status of older people (≥ 70 years) under consideration of the Transtheoretical model of health behavior change
MRC: Medical Research Council
SUS: System Usability Scale
TUG: Timed ‘Up and Go’ test
TTM: Transtheoretical Model of Behavior Change
MNA-SF: Mini Nutritional Assessment Short Form
BMI: Body Mass Index
SPPB: Short Physical Performance Battery
TC: Technology Commitment
