# Supplementary material for: Development and iterative optimization of an independently usable assistance system to assess, maintain and improve the nutritional and mobility status of older adults: an iterative usability study
Source: BMC Geriatr. 2026 Jan 6;26:264. doi: 10.1186/s12877-025-06950-1 (PMC12930998; doi:10.1186/s12877-025-06950-1)
Supplement: Supplementary file 1 — Additional file 1. [file 12877_2025_6950_MOESM1_ESM.docx]

**Appendix**

*Additional file 1: of the complete Test log of C3 with all elements used in the different sections of the test, their category, the precisely defined task and columns for noting the time needed for the task, the assistance required and the “Thinking Aloud” notes.*

| Subject ID:_________ | Test log C3 |  |  |
| --- | --- | --- | --- |

**Prologue:** I have just explained to you how to use all the elements of the station. However, please imagine that you received your briefing on the measurement and training station a week ago, and now you are expected to use the station over a period of several weeks. Today marks your first session after the initial briefing. I will guide you through the process and present various tasks. When I assign a task, I kindly request that you think out loud throughout. In other words, try to articulate your thoughts audibly. It’s essential that you also inform us if you find anything unclear or encounter difficulties. These insights will greatly assist us in further developing the measurement and training station. The goal of these tasks is not to test your abilities but rather to assess how well or poorly the elements we’ve developed perform and how we can enhance them. Do you have any questions for me?

|  | **Section** | **Category** | **Task** | **Time (sec.)** | **Assistance** | **„Thinking aloud“ Notes** |
| --- | --- | --- | --- | --- | --- | --- |
|  | Login with RFID Chip | Use of elements | You arrive at the measurement and training station and would like to check in. Please sign in using this bracelet. |  | None  Help 1*  Help 2**  Help 3 *** |  |
|  | Withings ScanWatch | Use of elements | Before commencing the tests, I request that you perform the pulse measurement using the watch. To do so, please access the instructions on the screen, follow them step by step, and read the pulse at the end.  CAUTION: Omit this step if patient has pacemaker |  | None  Help 1*  Help 2**  Help 3 *** |  |
|  | Touchscreen | Navigation simple | You want to start with the hand strength measurement today. How do you get to the screen where you can start the hand strength measurement? |  | None  Help 1*  Help 2**  Help 3 *** |  |
|  | Hand-strength-measurement | Use of elements | Please start the measurement of your hand-strength and follow the instructions.  CAUTION: Omit in advanced osteoporosis (Grade 3). Omit in the case of unhealed fractures, open wounds, or acute burn injuries on the forearm, wrist, or hand. |  | None  Help 1*  Help 2**  Help 3 *** |  |
|  | Touchscreen | Interpretation results  Interpretation results | What is displayed on the screen?  (An evaluation of the recently conducted hand-strength is displayed)  2.Could you please tell me how many kilograms you reached at maximum during the hand-strength-measurement? |  | None  Help 1*  Help 2**  Help 3 ***  None  Help 1*  Help 2**  Help 3 *** |  |
|  | Touchscreen | Navigation complex | Next, you should perform the “timed-up-and-go”- test. How do you get to the screen where you can start this assessment? |  | None  Help 1*  Help 2**  Help 3 *** |  |
|  | aTUG Chair | Use of elements | Please start the measurement and follow the instructions. |  | None  Help 1*  Help 2**  Help 3 *** |  |
|  | Touchscreen | Navigation simple | You have now completed the scheduled measurements for today and can start training. Please select the training device “Senso” on the screen and follow the instructions. |  | None  Help 1*  Help 2**  Help 3 *** |  |
|  | Senso | Use of elements | You want to play a training game called “Targets” today. Please start the game and follow the instructions.  CAUTION: Omit in advanced osteoporosis (Grade 3). |  | None  Help 1*  Help 2**  Help 3 *** |  |
|  | Touchscreen | Navigation complex | What do you need to select to display the results of todays training in the game “targets”? |  | None  Help 1*  Help 2**  Help 3 *** |  |
|  | Touchscreen | Interpretation results  Interpretation results | 1. Here you can see your results. Can you tell me what score you achieved today?  2. Let us pretend that you have played the game before. Did you achieve more points this time than in the last training session? |  | None  Help 1*  Help 2**  Help 3 ***  None  Help 1*  Help 2**  Help 3 *** |  |
|  | Touchscreen  (Navigation) | Navigation simple | How can you check how many spheres you missed during the training? |  | None  Help 1*  Help 2**  Help 3 *** |  |
| *Intermediate step: You have now completed your training for today. You remember that you wanted to fill out a questionnaire on nutrition to assess whether there is a risk of malnutrition. This questionnaire can be completed on the tablet you are using a s part of your participation in the study. Please go to the right desk, sit down, and take the tablet. You can activate it by pressing the small button on the right side of the tablet.* | | | | | | |
|  | Tablet  (Questionnaire) | Interpretation subject areas  Interpretation results  Navigation complex | You want to fill out the questionnaire first.  1.How can you answer the questions?  2.Can you tell me your score and what it means for you, specifically whether there is a risk of malnutrition or not?  3. Return to the nutrition start page. |  | None  Help 1*  Help 2**  Help 3 ***  None  Help 1*  Help 2**  Help 3 ***  None  Help 1*  Help 2**  Help 3 *** |  |
| *Intermediate step: You have already finished your training for today, completed the questionnaire, and now you feel like having a snack. You remember that you should also consume more protein to benefit even more from your training. The tablet includes recipe suggestions. You now want to search for a recipe.* | | | | | | |
|  | Tablet | Interpretation subject areas | In which of the displayed topic points do you think you would finds recipes for snacks? |  | None  Help 1*  Help 2**  Help 3 *** |  |
|  | Tablet | Navigation complex | Now try to find the recipe for the “Granola-Snack”. |  | None  Help 1*  Help 2**  Help 3 *** |  |
